# Erroneous bronchial transection after video assisted thoracoscopic surgery (VATS) pulmonary resection diagnosed with bronchoscopy: Case report

**DOI:** 10.1097/MD.0000000000018377

**Published:** 2019-12-16

**Authors:** Amit Borah, Steven Cocciardi, Ziad Boujaoude, Wissam Abouzgheib

**Affiliations:** Division of Pulmonary and Critical Care Medicine, Cooper Medical School at Rowan University, Camden, NJ.

**Keywords:** bronchoscopy, complications, interventional pulmonology, lung cancer

## Abstract

**Introduction:**

Early recognition of VATS-related complications is crucial for early interventions, treatments and better outcomes

**Patient concerns:**

Patient presented with post-obstructive pneumonia like symptoms 1 week after VATS pulmonary resection.

**Diagnosis:**

CT scan chest showed evidence of complete consolidation of the lobe where the pulmonary segmentectomy resection took place.

**Interventions:**

Diagnostic bronchoscopy confirmed the erroneous transection of the Superior Segment (SS) of Right Lower Lobe (RLL). Patient was then taken back for completion lobectomy and found with necrotic SS of RLL. This finding potentially could have caused significant complication if not recognized and treated early

**Outcomes:**

Patient recovered well after completion lobectomy and was discharged home several days later

**Conclusion:**

Erroneous bronchial transection should be suspected early in patients presenting with post-obstructive pneumonia after VATS pulmonary resection. CT scan chest and diagnostic bronchoscopy are the 2 main diagnostic tests

## 1 Introduction

Video Assisted Thoracoscopic Surgery (VATS) approaches are a mainstream technique within the discipline of thoracic surgery. This minimally invasive approach is currently employed for diagnostic and therapeutic lung surgery, as well as other procedures involving the mediastinum, chest wall, pericardium, esophagus and diaphragm. In general, VATS procedures are well tolerated and the prevalence of major intraoperative complications is around 1.5%.^[1–5]^ These include erroneous transections and injuries to broncho-vascular structures and neighboring organs which leads to additional major surgery and potential severe complications.^[1,2]^ Early recognition of these complications and immediate intervention are key in avoiding poor outcomes.

## 2 Case report

**Figure 1:**
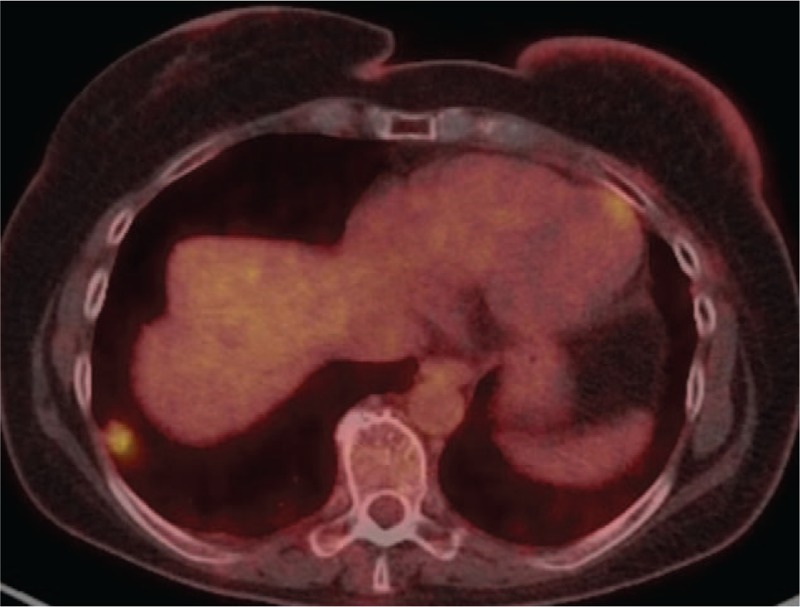
Pet/CT scan fused axial: RLL pet positive nodule.

**Figure 2:**
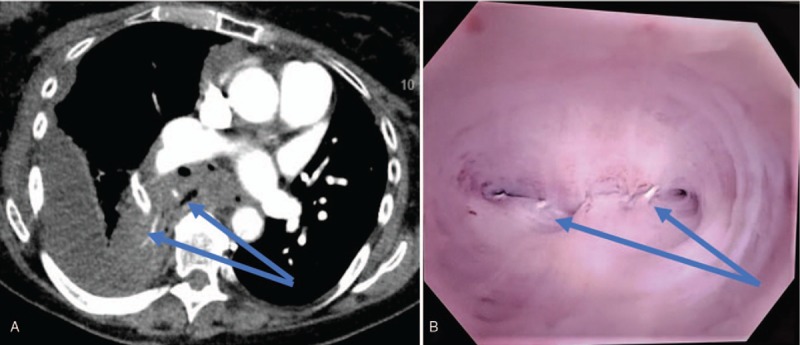
A. CT scan showing consolidated SS of RLL and proximal staple line; B. Bronchoscopic view showing erroneous transection of SS bronchus with obvious staples.

We present a 65 year old woman, with a history of prior left upper lobectomy for early stage non-small cell lung cancer, who underwent elective VATS for a highly suspicious right lower lobe lung nodule (Fig. 1). The nodule was wedge-resected and onsite pathology was positive for adenocarcinoma, with subsequent mediastinal lymphadenectomy revealing all negative results. The thoracic surgical team then proceeded with a basal segmental resection, reportedly sparing the superior segment (SS) of right lower lobe (RLL) and its vascular supply, with hopes of preserving all possible lung function given her history of prior left upper lobectomy. She presented one week later with fever, chills, loculated pleural effusion and apparent post-operative pneumonia. After a careful review of her current computed tomography (CT) chest imaging, we noted complete consolidation of SS of RLL and the presence of a surgical clip at the level of SS bronchus take-off. Diagnostic bronchoscopy revealed a staple line across the SS of RLL (Fig. 2). Our findings were discussed with the surgical team and the patient proceeded to the operating room where the right lower lobe superior segment was found to be pus-filled and necrotic. A completion right lower lobectomy with debridement and irrigation was performed and the patient did well post-operatively and was discharged several days later.

## 3 Discussion

In this case, diagnostic bronchoscopy led to an early recognition of erroneous bronchial transection which ultimately was treated with completion lobectomy and saved this patient significant morbidity. Had the bronchoscopy not been performed, her clinical and radiographic findings would have been blamed on severe pneumonia and broad-spectrum antibiotics would have been administered hoping for clinical improvement.

The erroneous bronchial transection has been reported as a major complication of VATS pulmonary resection ranging from 0.9 to 1.8%.^[1–5]^ In all cases, the fissure-last technique has been identified as a potential cause of this complication. In this technique, the surgeon dissects and transects the hilar structure first then proceeds to complete the fissure with a stapling device last.^[6,7]^ The fissure-last lobectomy requires advanced expertise and recognition of anatomical structures prior to attempting transection of the hilar structures. A ventilation test after clamping the targeted bronchus could also be helpful. The fissure-last technique could be challenging especially in right-lower lobectomy and when a ventilation test is neglected or not performed.^[6,7]^

Just like in this case, the evaluation of pneumonia early after VATS lung surgery should include a CT scan of the chest. Careful review of the operative report, identification and visualization of ipsilateral remaining proximal airways, and localization of surgical clips on imaging are crucial in diagnosing an erroneous bronchial transection of remaining neighboring lobes or segments to the resected part of the lung. If the CT scan shows an unexplained complete consolidation of any lobe or segment and bronchus cut-off sign with presence of proximal surgical clips, a diagnostic bronchoscopy is warranted with careful examination of the airways looking for erroneous transection. Discussion with the surgical team and aggressive surgical resection of the affected segment or lobe are the mainstay of treatment approach.

In summary, in patients presenting early after VATS pulmonary resection with signs and symptoms of pneumonia, CT scan chest with careful focus on airway patency is an important diagnostic test. Suspicious findings should be futher investigated with a diagnostic bronchoscopy to rule out an erroneous bronchial transection followed with surgical intervention if applicable

Patient has provided informed consent for publication of the case and accompanying images

## Author contributions

**Writing – original draft:** Amit Borah, Steven Cocciardi, Ziad Boujaoude, Wissam Abouzgheib.

**Writing – review & editing:** Amit Borah, Steven Cocciardi, Ziad Boujaoude, Wissam Abouzgheib.
